# Short-term and long-term effects of low serum bicarbonate level at admission in hospitalised patients

**DOI:** 10.1038/s41598-019-38892-1

**Published:** 2019-02-26

**Authors:** Sung Yoon Lim, Youngmi Park, Ho Jun Chin, Ki Young Na, Dong-Wan Chae, Sejoong Kim

**Affiliations:** 1 Department of Internal Medicine, Seoul National University Bundang Hospital, 82, Gumi-ro 173 Beon-gil, Bundang-gu, Seongnam-si, Gyeonggi-do 13620 Korea; 2 Medical Research Collaborating Center, Seoul National University Bundang Hospital, 82, Gumi-ro 173 Beon-gil, Bundang-gu, Seongnam-si, Gyeonggi-do 13620 Korea; 3 Department of Internal Medicine, Seoul National University College of Medicine, 103 Daehakro, Jongno-gu, Seoul 03080 Korea

## Abstract

Although low serum bicarbonate level is known to be associated with adverse outcomes in patients with chronic kidney injury, it is unclear whether low serum bicarbonate level is associated with the development of acute kidney injury (AKI). The purpose of our study was to determine whether serum bicarbonate levels at admission could be a risk factor for AKI development and mortality in hospitalised patients. We retrospectively enrolled 17,320 adult patients who were admitted to the academic teaching hospital from January 2013 to December 2013. Patients were divided into 2 groups based on the first measurement of serum bicarbonate level at admission. The incidence of AKI was higher in patients with low serum bicarbonate level than in those with normal serum bicarbonate level (8.0% vs. 4.1%). Low serum bicarbonate levels at admission were significantly associated with the development of AKI. In addition, low serum bicarbonate levels also independently predicted the 90-day mortality. Pre-existing low bicarbonate levels and subsequent development of AKI increased in-hospital mortality by 15 times compared with that in patients with normal bicarbonate levels and no AKI. Low serum bicarbonate levels may be associated with the development of AKI and high mortality in hospitalised patients.

## Introduction

Metabolic acidosis (MA), indicated by low serum bicarbonate levels, is a disorder that frequently develops in hospitalised patients. The presence of MA is strongly related to increased mortality, as it is implicated in multiple complications including cardiac dysfunction, hypotension, and increased risk of infection^1–3^.

Acute kidney injury (AKI) is also a common complication in hospitalised patients. Similar to MA, the presence of AKI is strongly related to mortality^4^. Once AKI develops, the chances of MA are increased. Decline in renal function causes an inability to excrete metabolic wastes and maintain proper acid-base balance, which results in MA^5^. Thus, clinical practice guidelines recommend the initiation of alkali therapy when the serum bicarbonate level is <22 mmol/L, although a recent Cochrane review has demonstrated that the benefit of sodium bicarbonate in AKI management is equivocal^6,7^.

Several observational studies have shown a crosstalk between MA and decline in renal function in patients with chronic kidney disease (CKD)^8^. A significant association of acidosis with all-cause mortality in patients with CKD has also been reported^9–11^. However, the impact of acidosis on the development of AKI has not yet been fully elucidated. In this study, we investigated whether lower serum bicarbonate level at the time of admission could predict the development of AKI, and whether AKI and low serum bicarbonate level have a combined effect on patient mortality.

## Results

A total of 17,320 patients were enrolled and divided into 2 groups according to the serum bicarbonate level. In the enrolled cohort, 25.91% (n = 4,488) were acidotic initially. During a median (interquartile range) hospital stay of 6.0 (3.0–10.0) days, AKI of all stages was detected in 882 (5.1%) patients, of whom 662 (3.8%) were in stage I and 220 (1.3%) were in stage II and stage III (Supplementary Table S1). Of the patients, 3.1% died of all causes within 90 days after admission. No patient died before the development of AKI.

### Baseline characteristics according to serum bicarbonate level

The patient demographics and clinical parameters at the time of admission are summarised in Table 1. Patients with low serum bicarbonate level were older than those with normal serum bicarbonate level, and more likely to have pre-existing comorbidities such as diabetes, hypertension, cardiovascular disease, and heart failure, except cancer. However, there was no significant difference in the Charlson comorbidity index score between the 2 groups.

**Table 1:** Baseline characteristics of patients with low serum bicarbonate and normal serum bicarbonate.

	Low serum bicarbonate (n = 4,488)	Normal serum bicarbonate (n = 12,832)	P
Age (years)	58.0 ± 18.6	58.0 ± 16.3	0.870
Male sex	2,136 (47.6%)	7,106 (55.4%)	0.000
Hypertension	301 (6.7%)	666 (5.2%)	0.000
Diabetes	261 (5.8%)	545 (4.2%)	0.000
Cardiovascular disease	302 (6.7%)	723 (5.6%)	0.005
Cancer	912 (20.3%)	3,382 (26.4%)	0.000
Charlson comorbidity index	5.7 ± 2.3	5.5 ± 2.0	0.000
Admission for elective surgical procedures	1,345 (30.0%)	5,108 (39.8%)	0.000
ICU stay history during the study period	878 (19.6%)	1,506 (11.7%)	0.000
RAS inhibitor	388 (8.6%)	872 (6.8%)	0.000
Diuretics	268 (6.0%)	485 (3.8%)	0.000
Body mass index (kg/m^2^)	23.9 ± 3.9	23.8 ± 3.6	0.035
Systolic BP (mmHg)	130.5 ± 22.7	130.6 ± 19.6	0.880
Diastolic BP (mmHg)	74.7 ± 14.5	75.8 ± 12.5	0.000
TWA-MAP (mmHg)	89.0 ± 9.1	87.5 ± 9.8	0.000
Use of vasopressors	121 (2.7%)	201 (1.6%)	0.000
Sodium (mmol/L)	138.1 ± 3.9	139.2 ± 3.0	0.000
White blood cells (10^9^/L)	9.6 ± 5.0	7.9 ± 5.8	0.000
Haemoglobin (g/L)	124 ± 23	129 ± 20	0.000
Platelet (10^9^/L)	214.5 ± 82.1	221.1 ± 79.2	0.000
C-reactive protein (mg/L)	56.19 ± 64.76	44.76 ± 55.24	0.000
Protein (g/L)	65 ± 9	66 ± 8	0.000
Albumin (g/L)	38 ± 6	40 ± 5	0.000
Total cholesterol (mmol/L)	4.5 ± 1.4	4.5 ± 1.1	0.113
Total bilirubin (µmol/L)	15.4 ± 29.1	13.7 ± 18.8	0.000
Serum creatinine (µmol/L)	61.0 ± 45.8	53.9 ± 22.9	0.000
eGFR (mL·min^−1^·1.73 m^−2^)	86.0 ± 32.8	91.8 ± 28.6	0.000

ICU, intensive care unit; RAS, renin-angiotensin system; BP, blood pressure; TWA-MAP, time-weighted average mean arterial pressure; eGFR, estimated glomerular filtration rate.

Values are expressed as mean ± standard deviation for continuous variables and n (%) for categorical variables.

*Incomplete data. The missing data rate was 8.9% in body mass index; 0.1% in systolic and diastolic BP; 1.2% in white blood cells, haemoglobin, and platelet; 45.6% in C-reactive protein; 2.2% in protein; 1.5% in albumin; 2.1% in cholesterol; and 2.2% in bilirubin.

The median serum bicarbonate levels in the low serum bicarbonate group and normal serum bicarbonate group were 20.0 (7–21) and 24.0 (22–29) mmol/L, respectively. Additionally, patients with low serum bicarbonate level at admission were more likely to have higher serum creatinine and to develop AKI. Compared with patients with normal serum bicarbonate level, those with low serum bicarbonate level had lower serum sodium, albumin, and estimated glomerular filtration rate (eGFR) at admission.

### Low serum bicarbonate, AKI, and mortality

**Figure 1:**
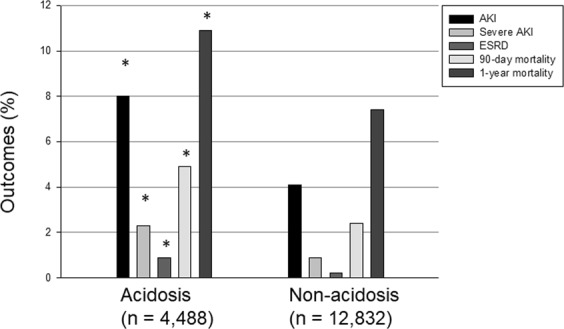
Clinical outcomes according to serum bicarbonate level. *P < 0.001 compared with the non-acidosis group. AKI, acute kidney injury; ESRD, end-stage renal disease.

A significantly higher rate of AKI development was observed in the low bicarbonate group than in the normal bicarbonate group (Fig. 1). Patients with AKI had lower serum bicarbonate levels than those without AKI. To evaluate the independent risk factors predicting the development of AKI, Cox regression analysis was performed (Supplementary Table S2, Table 2). Variables that were significant in univariate analysis, such as albumin, history of intensive care unit stay, hypertension, and diabetes mellitus, were included as adjusting covariates in multivariate analysis. As a result, lower serum bicarbonate level was independently associated with an increased risk of AKI development, with a hazard ratio (HR) of 1.574 (95% confidence interval [CI], 1.273–1.949; P < 0.001) in multivariate Cox proportional hazard regression analysis.

**Table 2:** Hazard ratio for the development of AKI and 90-day mortality in multivariable Cox proportional hazard regression.

	Acute kidney injury		90-Day mortality	
	HR (95% CI)	P	HR (95% CI)	P
Age (years)			1.031 (1.022–1.040)	<0.001
Male sex	1.428 (1.173–1.713)	<0.001		
Diabetes	1.971 (1.414–2.756)	<0.001		
Cardiovascular disease			1.591 (1.021–2.477)	0.040
Heart failure	2.444 (1.388–4.309)	0.002		
Cancer	1.262 (1.043–1.541)	0.020	5.852 (4.565–7.502)	<0.001
Diuretics	1.763 (1.255–2.484)	0.001	1.585 (1.140–2.205)	0.006
Albumin (g/L)	0.614 (0.489–0.772)	<0.001	0.406 (0.324–0.508)	<0.001
Total bilirubin (µmol/L)	1.083 (1.040–1.127)	<0.001	1.040 (1.012–1.070)	0.005
eGFR (mL·min^−1^·1.73 m^−2^)	1.013 (1.012–1.029)	<0.001		
ICU stay history	3.402 (2.762–4.173)	<0.001	1.940 (1.455–2.588)	<0.001
Admission for elective surgical procedures			0.188 (0.137–0.257)	<0.001
Low vs. normal serum bicarbonate	1.574 (1.273–1.949)	<0.001	1.302 (1.008–1.682)	0.043
Development of AKI	1.000		2.472 (1.900–3.217)	<0.001

HR, hazard ratio; CI, confidence interval; eGFR, estimated glomerular filtration rate; ICU, intensive care unit; AKI, acute kidney injury.

**Figure 2:**
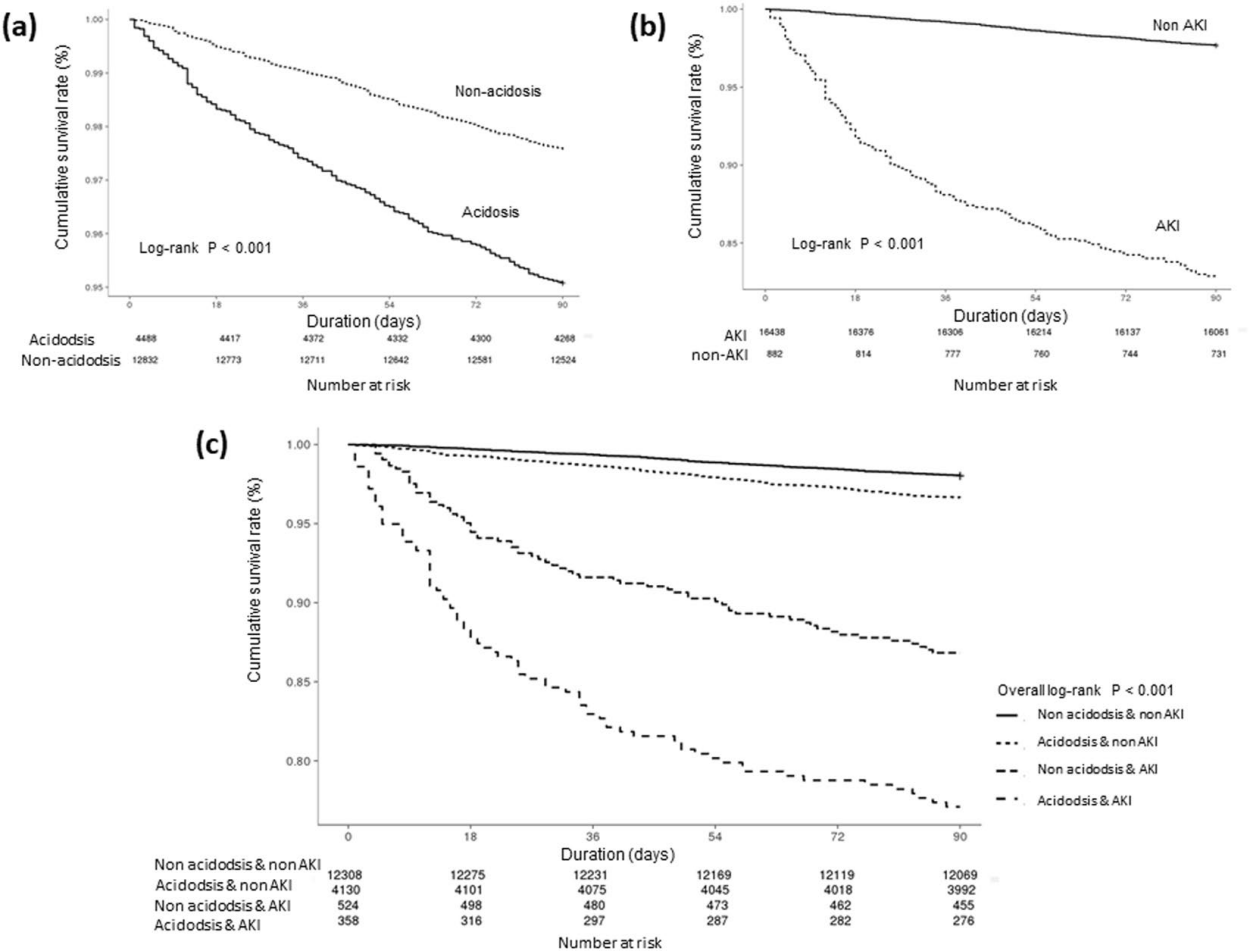
Cumulative survival rate according to serum bicarbonate level and acute kidney injury (AKI). (**a**–**c**) Show the survival curves of serum bicarbonate, AKI, and combined serum bicarbonate and AKI groups for the 90-day mortality, respectively. *And ^†^indicate P < 0.001 when compared with normonatraemic patients without AKI and hyponatraemic patients without AKI, respectively; ^‡^indicates P < 0.05 when compared with normonatraemic patients with AKI in the log-rank test.

**Figure 3:**
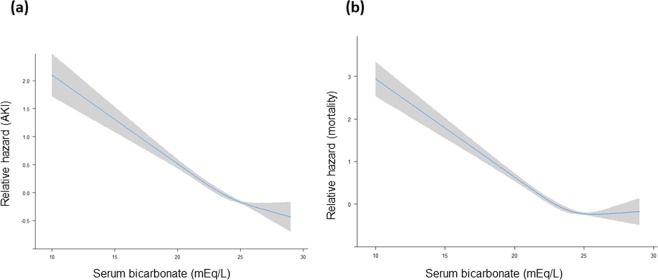
Relative hazards of acute kidney injury (AKI) development (**a**) and mortality (**b**) according to serum bicarbonate level in restricted cubic splines.

The low bicarbonate group also showed a statistically higher risk of severe AKI, end-stage renal disease at 1 year, and mortality of all causes within 90 days or 1 year after admission than the normal bicarbonate group (Fig. 1, Fig. 2a). Patients with AKI also had considerably higher risk of 90-day than those who did not develop AKI (Fig. 2b). As shown in Table 2, both low serum bicarbonate level and AKI were significant predictors of 90-day mortality in multivariate Cox proportional regression analysis (adjusted HR, 1.302; 95% CI, 1.008–1.682; P = 0.043 and adjusted HR, 2.472; 95% CI, 1.900–3.217; P < 0.001, respectively; Table 2). As low serum bicarbonate level was associated with the development of AKI, we assessed the interaction between low serum bicarbonate levels and AKI for mortality by using the relative excess risk due to interaction (Table 3). Compared with patients with normal serum bicarbonate level and without AKI, patients with low serum bicarbonate level and those with AKI had worse mortality (odds ratio [OR], 2.723; P < 0.001 and OR, 15.200; P < 0.001, respectively). In addition, patients in the low bicarbonate group with AKI had an increased risk of 90-day mortality by 18.863 times compared with patients in the normal bicarbonate group (P < 0.001). We also observed that the hazards of AKI and mortality decreased as the serum bicarbonate level increased in the restricted cubic regression models, and that higher serum bicarbonate level within the normal range seemed beneficial with respect to avoiding the development of AKI and mortality (Fig. 3).

**Table 3:** Interaction analysis between low serum bicarbonate and acute kidney injury for in-hospital mortality.

Low serum bicarbonate	AKI				OR (95% CI) for AKI (yes vs. no) within strata of low serum bicarbonate group
	No		Yes		
	n^*^	OR	n^*^	OR	
No Yes OR for low serum bicarbonate within strata of AKI group	244/12,308	1.0 (reference)	69/524	15.200	15.200
	82/358	2.723	139/4,130	18.863	6.927
		2.723		1.241	

Measure of interaction on additive scale (95% CI): RERI, 31.953 (8.622–55.284); AP, 0.491 (0.281–0.700); and SI, 1.993 (1.3076–3.0376).

Measure of interaction on the multiplicative scale: OR (95% CI) = 0.987 (0.508–1.921); P = 0.970.

*With/without mortality.

AKI, acute kidney injury; n, number; OR, odds ratio; CI, confidence interval; RERI, relative excess risk due to interaction; AP, attributable proportion due to interaction; SI, synergistic index.

## Discussion

In our large cohort study, we investigated the effect of low serum bicarbonate level at admission on the subsequent development of AKI in hospitalised patients. Patients with low serum bicarbonate level had an approximately 1.57-fold higher risk of AKI development than those with normal serum bicarbonate level. Low serum bicarbonate level also negatively affected survival and increased the risk of mortality in the presence of AKI. In addition, both AKI development and mortality proportionally increased with the severity of acidosis.

MA has been implicated in the pathogenesis of renal injury. The underlying exact mechanism for the association between MA and AKI is not clear; however, several possibilities can be considered on the basis of the results of previous animal and human studies. In MA, increased endothelin mediates urinary acidification and kidney injury^12,13^. Additionally, along with increased endothelin, excessive acid loading results in renin-angiotensin-aldosterone system activation^14^. In another study, MA was related to increased ammonia level, which, in turn, activated the alternative complement pathway and aggravated tubulointerstitial injury in an animal model of AKI^15,16^.

Furthermore, several observational studies have shown a clear relationship between MA and rapid decline in renal function in patients with CKD^8^. Although the mechanism of CKD progression due to MA cannot be directly applied to patients with AKI, reduced renal blood flow and inflammation may also be related to renal injury in patients with AKI. Several small trials suggested that the treatment of acidosis with oral alkali can slow the progression of kidney disease in patients with CKD^17^. In our study, patients treated with bicarbonate had higher rates of AKI development and mortality (data not shown). However, as patients treated with sodium bicarbonate had more severe disease and lower initial bicarbonate levels than patients who did not receive sodium bicarbonate, further well-designed trials are needed.

The higher number of AKI risk factors in patients with low serum bicarbonate level could affect the association between low serum bicarbonate level and AKI development. Therefore, we performed multivariate regression analysis, in which low serum bicarbonate level remained a significant risk factor of AKI development even after adjusting for baseline eGFR and other clinical and demographic factors. Consistent with these results, MA in kidney transplant recipients was a significant risk factor for graft failure and patient mortality, even after adjustment for eGFR^18^. Jung *et al*. also demonstrated that patients with low serum bicarbonate levels before cardiac surgery had a higher incidence of postoperative AKI independent of other risk factors for AKI development^19^. Similarly, recent observational studies found that lower serum bicarbonate levels predicted the development of AKI^5,20^, thereby supporting a close association between serum bicarbonate level and renal dysfunction.

There were some interactions between AKI and low serum bicarbonate level; thus, we analysed whether hospital-acquired AKI modified the effect of low serum bicarbonate level on short-term patient mortality. AKI can be further exacerbated by MA because of the reduction of renal blood flow and increase of inflammatory mediator release. Moreover, MA exacerbates renal injury, a phenomenon that was associated with a high nuclear factor-κB expression in an experimental AKI model^21^. These results could be a potential explanation for the synergistic interaction between MA and AKI. MA also affected long-term survival and increased mortality when AKI was concomitantly present.

Our study has several limitations. First, confounding factors originating from the retrospective design may have existed. Particularly, the different severities of illness on admission between the low and normal serum bicarbonate groups would be a major confounding variable. However, we excluded patients with community-acquired AKI. By excluding patients who had AKI before admission, we could clearly identify that MA preceded the development of AKI, reinforcing the relationship between MA and AKI. Moreover, the Charlson comorbidity index score was comparable between the 2 groups, and low serum bicarbonate level was a significant predictor of both AKI and 90-day mortality after adjusting for the severity of illness and other confounding variables. Second, our study was conducted in a single country and a single centre, limiting the generalisation of our findings. However, we collected data from a very large administrative cohort, and every variable was well measured; thus, there were few lost cases and laboratory data during the study period. Third, as our electronic medical records system does not keep records of the patients’ hourly urine output, we defined AKI only according to the serum creatinine criteria. Finally, measurements of arterial blood gas were not taken in all patients. Arterial pCO_2_ and pH were not determined, which leaves the possibility that the serum carbon dioxide levels may not represent the true serum bicarbonate levels in conditions such as respiratory alkalosis or sepsis. However, the prevalence of chronic obstructive pulmonary disease and heart failure was comparable between groups.

Low serum bicarbonate level in admitted patients is independently associated with the development of hospital-acquired AKI and long-term survival, and synergizes mortality in the presence of AKI. Therefore, patients with low serum bicarbonate level, which is easily detected using serum bicarbonate levels might need to be periodically monitored for serum creatinine level or urine output to check for AKI development.

## Methods

### Study population

We performed a single-centre, retrospective cohort study of patients aged 18 years or older who were admitted to Seoul National University Bundang Hospital from January 2013 to December 2013. This study initially included a total of 19,534 patients. We excluded patients who met the following exclusion criteria: (1) with community-acquired AKI; (2) with pre-existing end-stage renal disease that required renal replacement therapy before hospitalisation; and (3) with no available data or with high serum bicarbonate level (>31 mmol/L) within 2 days after admission. The remaining 17,320 patients were selected for the final analysis. The study received full approval from the Seoul National University Hospital institutional review board (IRB no. B-1408/264-102). The requirement for written informed consent was waived by the IRB, and this manuscript adheres to the applicable STROBE (STrengthening the Reporting of OBservational studies in Epidemiology) guidelines.

### Definitions and measurements

All data were collected from an electronic medical records database. The data included demographic data, comorbidities, and physiological data at admission. The time-weighted average mean arterial pressure (MAP) was calculated as the area under the curve of the MAP measurements divided by the total measurement time within 2 days after admission^22^. Total serum carbon dioxide levels, which are generally used as indirect measures of serum bicarbonate levels^18^, were determined using an electrode-based method (UniCel DxC 800; Beckman Coulter Inc., Brea, CA, USA). According to the first serum bicarbonate measurement within 2 days after admission, the patients were divided into 2 groups: the low bicarbonate group, which included patients whose serum bicarbonate level was <22 mmol/L, and the normal bicarbonate group, which included patients whose serum bicarbonate level was ≥22 mmol/L^6^.

Serum creatinine level was measured using the rate-blanked compensated kinetic alkaline picrate Jaffe method with an automatic analyser (TBA-200FR; Toshiba, Tokyo, Japan). The eGFR was calculated using the variable Modification of Diet in Renal Disease Study equation^23^. Baseline creatinine was defined as the lowest value within 6 months before the index admission or the calculated value from the Modification of Diet in Renal Disease study equation, assuming that the baseline glomerular filtration rate is 75 mL·min^−1^·1.73 m^−2^ if creatinine was not available^24^. AKI was defined as an increase in serum creatinine level of 26.5 mmol/L (0.3 mg/dL) greater than the baseline value or a 1.5-fold higher value than the baseline level determined during the hospital stay^25^. We defined AKI solely according to changes in measured serum creatinine values because urine output data were not consistently available for all inpatients. We defined severe AKI as stage 2 and 3 AKI based on the Kidney Disease: Improving Global Outcomes classification^25^. If the first creatinine level at the index admission met the criteria for AKI, we defined it as a community-acquired AKI. The development of end-stage renal disease was determined from the registry database of the Korean Society of Nephrology. Patient mortality was determined from the death certificates, and 1-year mortality was determined from the database of the Ministry of Interior.

### Statistical analysis

Values were expressed as mean ± standard deviation or median (interquartile range) for continuous variables and as percentage for categorical variables. The difference was analysed using Student’s t-test for continuous variables and the chi-square test for categorical variables. For the estimated survival, the Kaplan-Meier method was employed, and the statistical significance was calculated using the log-rank test. A Cox proportional hazards regression analysis was performed to determine the association between low serum bicarbonate level and AKI or mortality. We fitted a multivariate Cox regression model with significant variables (P < 0.05) from the univariate Cox regression analysis. Further, the probability of AKI and mortality in relation to serum bicarbonate level at admission was presented using restricted cubic splines. For the estimated survival, the Kaplan-Meier method was employed, and the statistical significance was calculated using the log-rank test. The additive interaction was assessed using the relative excess risk due to interaction, attributable proportion due to interaction, and synergistic index^26^, with R statistics (version 3.0.3; R Foundation for Statistical Computing, Vienna, Austria). Unless specified, all analyses were performed using SPSS Statistics (version 20; IBM, Armonk, NY, USA).

## Supplementary information

Supplementary Table

## References

[1] Gunnerson KJ, Saul M, He S, Kellum JA (2006). Lactate versus non-lactate metabolic acidosis: a retrospective outcome evaluation of critically ill patients. Critical care (London, England).

[2] Kraut JA, Madias NE (2010). Metabolic acidosis: pathophysiology, diagnosis and management. Nature reviews. Nephrology.

[3] Masevicius FD (2017). Relationship of at Admission Lactate, Unmeasured Anions, and Chloride to the Outcome of Critically Ill Patients. Critical care medicine.

[4] Nash K, Hafeez A, Hou S (2002). Hospital-acquired renal insufficiency. American journal of kidney diseases: the official journal of the National Kidney Foundation.

[5] Gujadhur A (2015). Serum bicarbonate may independently predict acute kidney injury in critically ill patients: An observational study. World journal of critical care medicine.

[6] Khwaja A (2012). KDIGO clinical practice guidelines for acute kidney injury. Nephron. Clinical practice.

[7] Hewitt, J., Uniacke, M., Hansi, N. K., Venkat-Raman, G. & McCarthy, K. Sodium bicarbonate supplements for treating acute kidney injury. *The Cochrane database of systematic reviews*, Cd009204, 10.1002/14651858.CD009204.pub2 (2012). 10.1002/14651858.CD009204.pub2 PMC6481784 22696382

[8] Dobre M (2013). Association of serum bicarbonate with risk of renal and cardiovascular outcomes in CKD: a report from the Chronic Renal Insufficiency Cohort (CRIC) study. American journal of kidney diseases: the official journal of the National Kidney Foundation.

[9] Kovesdy CP (2012). Metabolic acidosis and kidney disease: does bicarbonate therapy slow the progression of CKD?. Nephrology, dialysis, transplantation: official publication of the European Dialysis and Transplant Association - European Renal Association.

[10] Menon V (2010). Serum bicarbonate and long-term outcomes in CKD. American journal of kidney diseases: the official journal of the National Kidney Foundation.

[11] Navaneethan SD (2011). Serum bicarbonate and mortality in stage 3 and stage 4 chronic kidney disease. Clinical journal of the American Society of Nephrology: CJASN.

[12] Wesson DE, Simoni J (2010). Acid retention during kidney failure induces endothelin and aldosterone production which lead to progressive GFR decline, a situation ameliorated by alkali diet. Kidney international.

[13] Wesson DE, Nathan T, Rose T, Simoni J, Tran RM (2007). Dietary protein induces endothelin-mediated kidney injury through enhanced intrinsic acid production. Kidney international.

[14] Ng HY, Chen HC, Tsai YC, Yang YK, Lee CT (2011). Activation of intrarenal renin-angiotensin system during metabolic acidosis. American journal of nephrology.

[15] Nath KA, Hostetter MK, Hostetter TH (1991). Increased ammoniagenesis as a determinant of progressive renal injury. American journal of kidney diseases: the official journal of the National Kidney Foundation.

[16] Pangburn MK, Schreiber RD, Muller-Eberhard HJ (1981). Formation of the initial C3 convertase of the alternative complement pathway. Acquisition of C3b-like activities by spontaneous hydrolysis of the putative thioester in native C3. The Journal of experimental medicine.

[17] Chen W, Abramowitz MK (2014). Metabolic acidosis and the progression of chronic kidney disease. BMC nephrology.

[18] Park S (2017). Metabolic Acidosis and Long-Term Clinical Outcomes in Kidney Transplant Recipients. Journal of the American Society of Nephrology: JASN.

[19] Jung SY (2016). Preoperative Low Serum Bicarbonate Levels Predict Acute Kidney Injury After Cardiac Surgery. Medicine.

[20] Hu J (2017). Metabolic acidosis as a risk factor for the development of acute kidney injury and hospital mortality. Experimental and therapeutic medicine.

[21] Magalhaes PA (2016). Metabolic acidosis aggravates experimental acute kidney injury. Life sciences.

[22] Octavio JA (2010). Time-weighted vs. conventional quantification of 24-h average systolic and diastolic ambulatory blood pressures. Journal of hypertension.

[23] Levey AS (1999). A more accurate method to estimate glomerular filtration rate from serum creatinine: a new prediction equation. Modification of Diet in Renal Disease Study Group. Annals of internal medicine.

[24] Manjunath G, Sarnak MJ, Levey AS (2001). Prediction equations to estimate glomerular filtration rate: an update. Current opinion in nephrology and hypertension.

[25] Section 2: AKI Definition. *Kidney international supplements* **2**, 19–36, 10.1038/kisup.2011.32 (2012). 10.1038/kisup.2011.32 PMC4089595 25018918

[26] Rothman KJ (1974). Synergy and antagonism in cause-effect relationships. American journal of epidemiology.

