# Supplementary material for: Short-term and long-term effects of low serum bicarbonate level at admission in hospitalised patients
Source: Sci Rep. 2019 Feb 26;9:2798. doi: 10.1038/s41598-019-38892-1 (PMC6391433; doi:10.1038/s41598-019-38892-1)
Supplement: Supplementary file 1 — Supplementary Table [file 41598_2019_38892_MOESM1_ESM.docx]

**Short-term and long-term effects of low serum bicarbonate level at admission in hospitalised patients**

**Sung Yoon Lim^1^, Youngmi Park^2^, Ho Jun Chin^1^, Ki Young Na^1^, Dong-Wan Chae^1^, Sejoong Kim^1^***

**^1^Department of Internal Medicine, Seoul National University Bundang Hospital, 82, Gumi-ro 173 Beon-gil, Bundang-gu, Seongnam-si, Gyeonggi-do, 13620, Korea**

**^2^Medical Research Collaborating Center, Seoul National University Bundang Hospital, 82, Gumi-ro 173 Beon-gil, Bundang-gu, Seongnam-si, Gyeonggi-do, 13620, Korea**

***Corresponding author**

**Sejoong Kim, MD**

**82, Gumi-ro 173 Beon-gil, Bundang-gu, Seongnam-si, Gyeonggi-do, 13620, Korea; Tel.: 031-787-7051; Fax: 031-787-; E-mail: sejoong2@snu.ac.kr**

**Supplementary Table S1. AKI stage and timing**

|  | **Total (N = 17,320)** | **Low serum bicarbonate (n = 4,488)** | **Normal serum bicarbonate (n = 12,832)** | **P** |
| --- | --- | --- | --- | --- |
| KDIGO |  |  |  | 0.000 |
| Stage 1 | 662 (3.8%) | 255 (5.7%) | 407 (3.2%) |  |
| Stage 2 | 131 (0.8%) | 57 (1.3%) | 74 (0.6%) |  |
| Stage 3 | 89 (0.5%) | 46 (1.0%) | 43 (0.3%) |  |
| Haemodialysis | 36 (0.2%) | 25 (0.6%) | 11 (0.1%) | 0.000 |
| CRRT | 47 (0.3%) | 26 (0.6%) | 21 (0.2%) | 0.000 |
| Hospital day of AKI diagnosis | 8.7 ± 10.1 | 7.4 ± 9.1 | 9.7 ± 10.7 | 0.001 |

KDIGO, Kidney Disease: Improving Global Outcomes; CRRT, continuous renal replacement therapy; AKI, acute kidney injury.

Values are expressed as mean ± standard deviation for continuous variables and n (%) for categorical variables.

**Supplementary Table S2. Hazard ratio for the development of AKI and 90-day mortality in univariate Cox proportional hazard regression**

|  | | Acute kidney injury | | | 90-Day mortality | |
| --- | --- | --- | --- | --- | --- | --- |
|  | | HR (95% CI) | P | HR (95% CI) | | P |
| Age (years) | | 1.03 (1.03–1.03) | <0.001 | 1.06 (1.05–1.06) | | <0.001 |
| Male sex | | 1.31 (1.14–1.50) | <0.001 | 1.59 (1.33–1.90) | | <0.001 |
| Hypertension | | 1.52 (1.18–1.95) | 0.001 | 1.15 (0.81–1.63) | | 0.422 |
| Diabetes | | 2.36 (1.87–2.98) | <0.001 | 1.36 (0.95–1.93) | | 0.089 |
| Cardiovascular disease | | 1.42 (1.11–1.83) | 0.006 | 1.59 (1.18–2.14) | | 0.002 |
| Heart failure | | 7.94 (5.55–11.35) | <0.001 | 3.65 (2.22–6.00) | | <0.001 |
| Cancer | | 1.50(1.29–1.73) | <0.001 | 5.12 (4.30–6.10) | | <0.001 |
| RAS inhibitor | | 1.75 (1.41–2.16) | <0.001 | 0.95 (0.68–1.33) | | 0.765 |
| Diuretics | | 3.25 (2.61–4.05) | <0.001 | 3.03 (2.33–3.94) | | <0.001 |
| Body mass index (kg/m^2^) | | 0.96 (0.94–0.98) | <0.001 | 0.84 (0.81–0.86) | | <0.001 |
| TWA-MAP (mmHg) | | 1.00 (0.99–1.01) | 0.717 | 0.98 (0.97–0.99) | | 0.001 |
| Use of vasopressors | | 7.32 (5.67–9.46) | <0.001 | 2.66 (1.78–3.97) | | <0.001 |
| ICU stay history during the study period | | 4.28 (3.71–4.94) | <0.001 | 2.29 (1.89–2.78) | | <0.001 |
| Admission for elective surgical procedure | | 1.25 (1.09–1.44) | 0.001 | 0.28 (0.22–0.36) | | <0.001 |
| Sodium (mmol/L) | | 0.91 (0.89–0.92) | <0.001 | 0.87 (0.86–0.89) | | <0.001 |
| White blood cells (10^9^/L) | | 1.02 (1.01–1.02) | <0.001 | 1.02 (1.01–1.02) | | <0.001 |
| Haemoglobin (g/L) | | 0.82 (0.79–0.85) | <0.001 | 0.73 (0.70–0.75) | | <0.001 |
| Platelet (10^9^/L) | | 1.00 (1.00–1.00) | 0.485 | 1.00 (1.00–1.00) | | 0.952 |
| C-reactive protein (mg/L) | | 1.03 (1.02–1.04) | <0.001 | 1.06 (1.05–1.07) | | <0.001 |
| Protein (g/L) | | 0.69 (0.64–0.75) | <0.001 | 0.65 (0.59–0.71) | | <0.001 |
| Albumin (g/L) | | 0.38 (0.34–0.43) | <0.001 | 0.25 (0.22–0.28) | | <0.001 |
| Total cholesterol (mmol/L) | | 0.99 (0.99–0.99) | <0.001 | 0.99 (0.99–0.99) | | <0.001 |
| Total bilirubin (µmol/L) | | 1.16 (1.13–1.20) | <0.001 | 1.16 (1.14–1.18) | | <0.001 |
| eGFR (mL⋅min^-1^⋅1.73 m^-2^) | | 1.01 (1.01–1.01) | <0.001 | 1.00 (1.00–1.01) | | 0.002 |
| Low vs. normal serum bicarbonate | 2.37 (2.03–2.77) | | <0.001 | 2.69 (2.24–3.23) | | <0.001 |
| Development of AKI | 1.00 | |  | 8.18 (6.78–9.88) | | <0.001 |

HR, hazard ratio; CI, confidence interval; RAS, renin-angiotensin system; TWA-MAP, time-weighted average mean arterial pressure; eGFR, estimated glomerular filtration rate.
